# Assessment of the effect of vacuum-formed retainers and Hawley retainers on periodontal health: A systematic review and meta-analysis

**DOI:** 10.1371/journal.pone.0253968

**Published:** 2021-07-09

**Authors:** Bowen Li, Yifeng Xu, Cailian Lu, Zhenheng Wei, Yongyue Li, Jinghui Zhang

**Affiliations:** 1 Department of Stomatology, Inner Mongolia Medical University, Hohhot, Peoples R, China; 2 Department of Orthodontics, Inner Mongolia Medical University Third Affiliated Hospital, Baotou, Peoples R, China; Virginia Commonwealth University, UNITED STATES

## Abstract

**Background:**

Recently, increasing attention has been paid to the periodontal health of orthodontic patients in the maintenance stage in clinical practice. The focus of this meta-analysis was to compare the effects of vacuum-formed retainers (VFR) and Hawley retainers (HR) on periodontal health, in order to provide a reference for clinical selection.

**Methods:**

From the establishment of the database until November 2020, a large number of databases were searched to find relevant randomized control trials, including the Cochrane Library databases, Embase, PubMed, Medline via Ovi, Web of Science, Scopus, Grey Literature in Europe, Google Scholar and CNKI. Related literature was manually searched and included in the analysis. Two researchers screened the literature according to relevant criteria. The size of the effect was determined using RevMan5.3 software, and the mean difference and 95% confidence intervals (CI) were used to estimate the results using a random effects model.

**Results:**

This meta-analysis included six randomized controlled trials involving 304 patients. The results of the meta-analysis showed that there was no statistical difference in sulcus probing depth status between the VFR group and the HR group, including at 1, 3, and 6 months. Compared with the VFR group, the HR group showed a lower gingival index at 1 month (mean difference = 0.12, 95%CI: 0.06 to 0.19) and 3 months (mean difference = 0.11, 95%CI: 0.06 to 0.17), while there was no statistically significant difference at 6 months (mean difference = 0.10, 95%CI: -0.07 to 0.27). The plaque index of the HR group also showed a good state at 1 month (mean difference = 0.06, 95%CI: 0.01 to 0.12), 3 months (mean difference = 0.12, 95%CI: 0.08 to 0.16), and 6 months (mean difference = 0.19, 95%CI: 0.09 to 0.29). Subgroup analysis of PLI showed that when all teeth were measured, PLI status was lower in the HR group at 6 months (mean difference = 0.32, 95%CI: 0.18 to 0.46). PLI status was also low for the other teeth group (mean difference = 0.15, 95%CI: 0.08 to 0.22).

**Conclusion:**

Our meta-analysis showed that patients using the Hawley retainer had better periodontal health compared with those using vacuum-formed retainers. However, more research is needed to look at the periodontal health of patients using these two retainers.

## 1. Introduction

After orthodontic treatment, the time required for bone reconstruction to reach a stable state and for the adaptive changes of muscles and soft tissues around dentition after treatment, is longer than the time required for simple tooth alignment. Therefore, the use of retainers is critical for obtaining a lasting and stable effect [1, 2]. After the orthodontic treatment, the retainer should be routinely worn for more than 2 years, and adults may even need to wear it for life [3, 4]. The retainer itself favors plaque retention (a plaque retention area) and plaque aggregation. Secondly, the wearing of the retainer hinders the self-cleaning effect of the oral cavity and the implementation of oral cleaning measures. Long-term retention of plaque will cause inflammation in periodontal tissues, which will lead to the destruction of periodontal hard and soft tissues and even the resorption of alveolar bone [5]. Therefore, the influence of retainers on periodontal health should not be ignored. At present, the most commonly used clinical mobile retainers are vacuum-formed retainers and Hawley retainers. The Hawley retainer, which consists of a resin base and a clasp placed on the lip of the tooth, has been widely used in clinical practice since 1919. In recent years, the vacuum-formed retainer has gradually been favored by orthodontists and patients because of its simple production, low cost, and good aesthetics [6].

Some studies [7–9] have suggested that, in order to reduce the risk of recurrence, orthodontic retainers must be worn for a long time, some even need to be worn for nearly 24 hours a day, and the long-term contact of the retainer with the tissue in the oral cavity may promote plaque accumulation, which can affect the health of periodontal tissue. This situation is especially problematic at night, even if the retainer is properly cleaned [10, 11]. However, the effect of these two types of retainers on periodontal health has not been determined. In recent years, numerous studies on the periodontal health of patients using different retainers have been conducted. Eroglu et al. [12] investigated the periodontal clinical parameters and found no statistical difference in plaque index (PLI), gingival index (GI), probing bleeding, and probing depth between users of Hawley retainer and those of vacuum-formed retainers. However, Zhang and Wang [13] found that, compared with users of Hawley retainer, patients wearing a vacuum-formed retainer were more prone to gingival inflammation.

Since this clinical issue is still controversial, we aimed to perform a meta-analysis of the existing literature on the influence of these two types of retainers on periodontal health, hoping to provide objective clinical guidance and a theoretical basis for the clinical selection of appropriate retainers.

## 2. Methods

### 2.1 Literature search and screening

This meta-analysis was registered at the International Prospective Register of Systematic Reviews (number CRD42020222090). This meta-analysis is reported in accordance with the Preferred Reporting Items for Systematic Reviews and Meta-Analyses (PRISMA) Statement [14].

From the establishment of the database to November 2020, a large number of databases were retrieved, including Cochrane Library Databases, Embase, PubMed, Medline via Ovi, Web of Science, Scopus, Grey Literature in Europe, Google Scholar and CNKI. We searched for unpublished articles through ClinicalTrials.gov (www.clinicaltrials.gov) and the National Research Register. The search was conducted using medical subject terms (MESH) and free words. PubMed’s search strategy is described in Table 1. Cochrane Library Database’s search strategy is described in Table 2. Other databases used revised search strategies with the assistance of a librarian. Due to language limitations, only articles published in Chinese or English journals were included in this meta-analysis. Because this analysis is based on previously published studies, moral consent and patient consent were not required.

**Table 1:** Search strategy for PubMed.

Literature search was conducted up to 11/2020 PubMed results		
#1	(((((((((Retainers, Orthodontic) OR (Orthodontic Retainer)) OR (Retainer, Orthodontic)) OR (HAWLEY retainer)) OR (Hawley orthodontic retainer)) OR (vacuum-formed retainers)) OR (Thermoplastic Retainers)) OR (Essix retainers)) OR (removable retainers)) OR ("Orthodontic Retainers"[Mesh])	1774
#2	(((((((((((((((((Index, Periodontal) OR (Indices, Periodontal)) OR (Periodontal Indices)) OR (Periodontal Indexes)) OR (Indexes, Periodontal)) OR (Community Periodontal Index of Treatment Needs)) OR (CPITN)) OR (Bleeding on Probing, Gingival)) OR (Gingival Bleeding on Probing)) OR (Gingival Index)) OR (Gingival Indices)) OR (Index, Gingival)) OR (Indices, Gingival)) OR (Gingival Indexes)) OR (Indexes, Gingival)) OR ("Periodontal Index"[Mesh])) OR (Probing Depth)) OR (Sulcus Probing Depth)	28523
#3	(((((((((randomized controlled trial [pt]) OR controlled clinical trial [pt]) OR randomized [tiab]) OR placebo [tiab]) OR drug therapy [sh]) OR randomly [tiab]) OR trial [tiab]) OR groups [tiab])) NOT ((animals [mh] NOT humans [mh]))	4293764
#4	#1 AND #2 AND #3	31

**Table 2:** Search strategy for Cochrane Library database.

Literature search was conducted up to 11/2020 Cochrane results		
#1	MeSH descriptor: [Orthodontic Retainers] explode all trees	93
#2	MeSH descriptor: [Periodontal Index] explode all trees	1991
#3	(Retainers, Orthodontic) OR (Orthodontic Retainer) OR (Retainer, Orthodontic) OR (after orthodontic treatment)	2129
#4	#1 or #3	1860
#5	(Index, Periodontal) OR (Indices, Periodontal) OR (Indexes, Periodontal) OR (Community Periodontal Index of Treatment Needs) OR (CPITN):ti,ab,kw (Word variations have been searched)	5290
#6	(Bleeding on Probing, Gingival) OR (Gingival Bleeding on Probing) OR (Gingival Index) OR (Gingival Indices) OR (Gingival Indexes) (Word variations have been searched)	5090
#7	(Probing Depth) OR (Sulcus Probing Depth)	4414
#8	#2 or #5 or #6 or #7	7069
#9	#4 and #8	332

### 2.2 Inclusion criteria

Randomized controlled trials (RCTs) have compared the periodontal health of patients undergoing orthodontic maintenance using Hawley retainer or vacuum-formed retainer. The studies included patients diagnosed with malocclusion who required an orthodontic retainer for the maintenance of the outcome after treatment with a fixed appliance and with no history of systemic disease or periodontitis. Professional oral hygiene instruction was provided for all subjects. Vacuum-formed retainer and Hawley retainer were the types of intervention employed in the studies.

### 2.3 Exclusion criteria

We excluded non-randomized controlled studies, non-clinical studies, such as pigs or dogs. Data were not available for use in meta-analysis. Studies whose data was unavailable even after contacting the authors were excluded from the meta-analysis. Patients with periodontitis were excluded. Participants with poor oral hygiene, the ones who used antibiotics or hormones 1 month before joining the study, pregnant patients and patients who presented risk factors for periodontal disease (diabetes and tobacco smoking). Patients with underlying diseases or other maxillofacial abnormalities were also excluded.

### 2.4 Observation outcome

PLI, GI, and SPD were analyzed. The results of the above parameters at T1 (1 month), T2 (3 months), and T3 (6 months) were evaluated in this meta-analysis.

### 2.5 Data collection

We selected the eligible RCT studies according to the standard procedures used to identify articles, including the screening of titles and abstracts, the selection of eligible studies for evaluation, and the extraction of data from included studies. Data were selected, extracted, and evaluated independently by two authors (BL and YX) to determine whether they met the inclusion criteria for this meta-analysis. In case of disagreement, a third reviewer (JZ) assisted in the judgment.

### 2.6 Quality assessments

The two reviewing authors (BL and YX) assessed the bias of the RCT trials using the Cochrane Risk of Bias tool and independently assessed the overall risk of bias in each study using a specific table as reference. The Cochrane Handbook recommends that these specific areas be assessed for systematic evaluation. If all areas were considered to be low, the study was judged to have a low risk of bias. If at least one domain was judged to be unclear, a moderate risk of bias was considered. A high risk of bias was considered in research studies with at least one area of high risk. If no agreement was reached, the third author (JZ) was consulted.

### 2.7 Data extraction and management

The following information was used by the two reviewing authors (BL and YX), who developed a data retrieval plan and recorded basic information using a data extraction form, respectively, as follows: author’s name, publication date, study design, authors’ nationality, sample size, average age of patients, intervention, outcome indicators, and times of measurement. The third reviewing author (JZ) resolved all differences. All citations from the database were imported into Endnote X9 (Clarivate Analytics), which uses the Find Duplicates feature to identify and remove duplicates.

### 2.8 Statistical analysis

Statistical analysis was performed using RevMan 5.3 software. The chi-square test was used to assess heterogeneity. I2 < 50%, indicates the homogeneity of included studies; I2>50% represents a high degree of heterogeneity using a random effects model. The 95% confidence interval (CI) estimates and results for each variable were visualized in a forest plot. For each outcome indicator with significant heterogeneity, we screened the source of heterogeneity through subgroup analysis or sensitivity analysis and excluded one study at a time. If the studies were less than ten, no funnel plots were used for bias assessment.

## 3. Results

### 3.1 Study selection and characteristics

**Fig 1:**
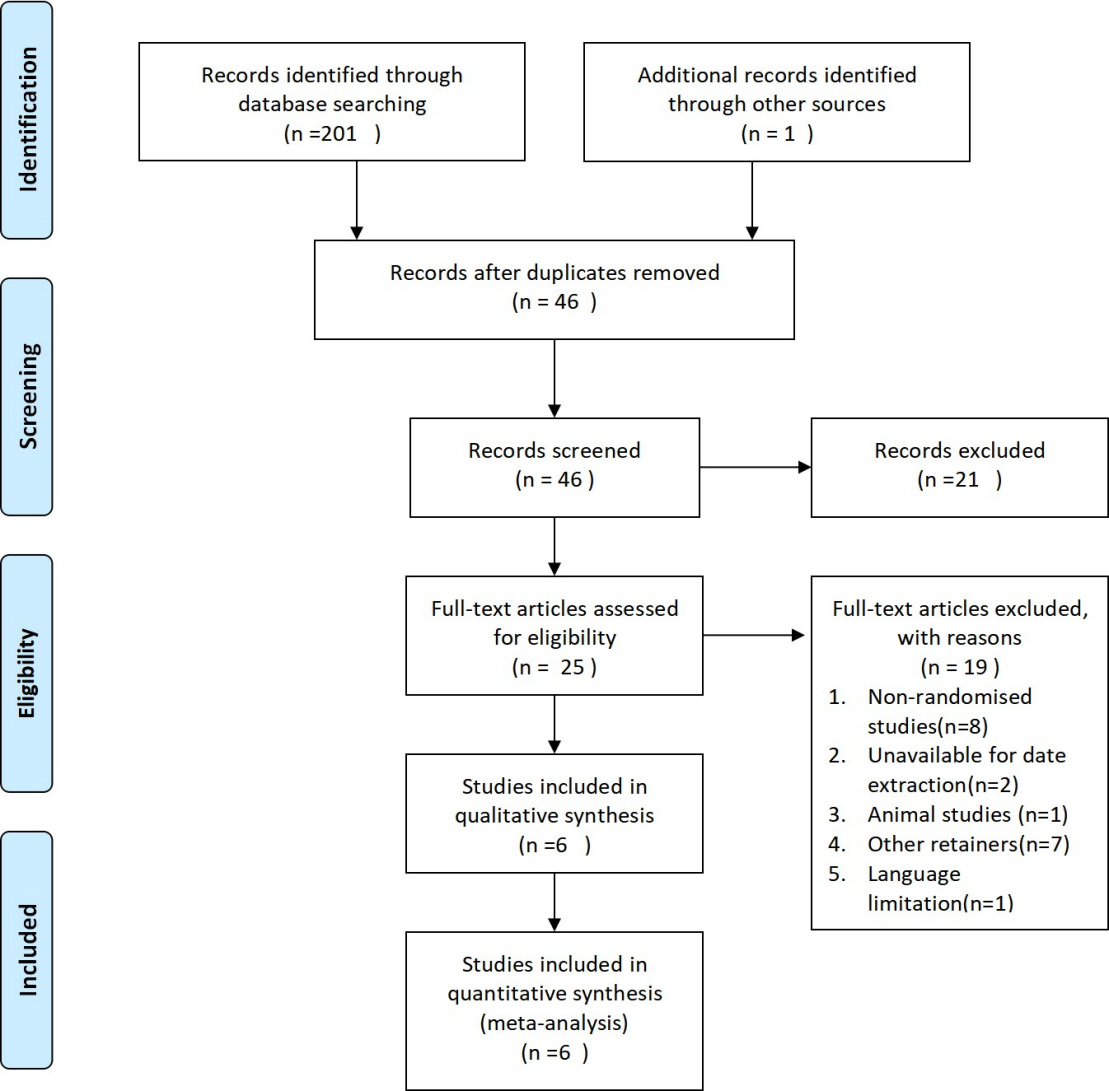
PRISMA 2009 flow diagram.

A total of 202 studies were selected in a preliminary search, with 156 duplicates. After an initial screening of titles and abstracts, 15 studies were excluded, and we excluded six articles after reading the methods. A total of 25 full-text studies were retrieved, and 19 studies were excluded based on relevant criteria, including 1 study in French, which was excluded due to language limitations. Finally, a total of six studies involving 304 patients were included in the meta-analysis. Among these patients, 152 were in the VFR group and 152 were in the Hawley group. The flow chart for the study selection is shown in Fig 1. The main information contained in each study is shown in Table 3.

**Table 3:** Characteristics of the included studies.

Author(s)/year	Study design	Country	Sample size	Average age	Intervention	Outcome measures	Time measures	Main conclusion
Eroglu, A. K [12] 2019	RCT	Turkey	Group A 15 Group B 15	15.2±2.1 y	Group A VFR Group B Hawley	GI,PLI,SPD,	T1, T2	Hawley retainers ≈ vacuum-formed retainers(p>0.05)
Moslemzadeh, S. H [15] 2018	RCT	Iran	Group A 22 Group B 22	18.8y	Group A maxilla VFR Group B maxilla Hawley	GI	T3	Hawley retainers ≈ vacuum-formed retainers(p>0.05)
Wang Hua [16] 2015	RCT	China	Group A 16 Group B 16	16.9y	Group A VFR Group B Hawley	GI,PLI,SPD	T3	Hawley retainers > vacuum-formed retainers(p<0.05)
Yang Yong [17] 2008	RCT	China	Group A 28 Group B 28	14.5y	Group A VFR Group B Hawley	GI,PLI	T1,T2,T3	Hawley retainers > vacuum-formed retainers(p<0.05)
Zhang Baoru [13] 2003	RCT	China	Group A 31 Group B 31	15.7y	Group A VFR Group B Hawley	GI,PLI,SPD	T1, T2	Hawley retainers > vacuum-formed retainers(p<0.05)
Zhou Yan [18] 2009	RCT	China	Group A 40 Group B 40	17.3y	Group A VFR Group B Hawley	GI,PLI,SPD	T1, T2, T3	Hawley retainers < vacuum-formed retainers(p<0.05)

RCT = randomized controlled trials, VFR = vacuum-formed retainer, HR = Hawley retainer, GI = gingival index, PLI = plaque index, SPD = sulcus probing depth, T1 = 1 month, T2 = 3 months, T3 = 6 months.

### 3.2 Risk of bias assessment

**Fig 2:**
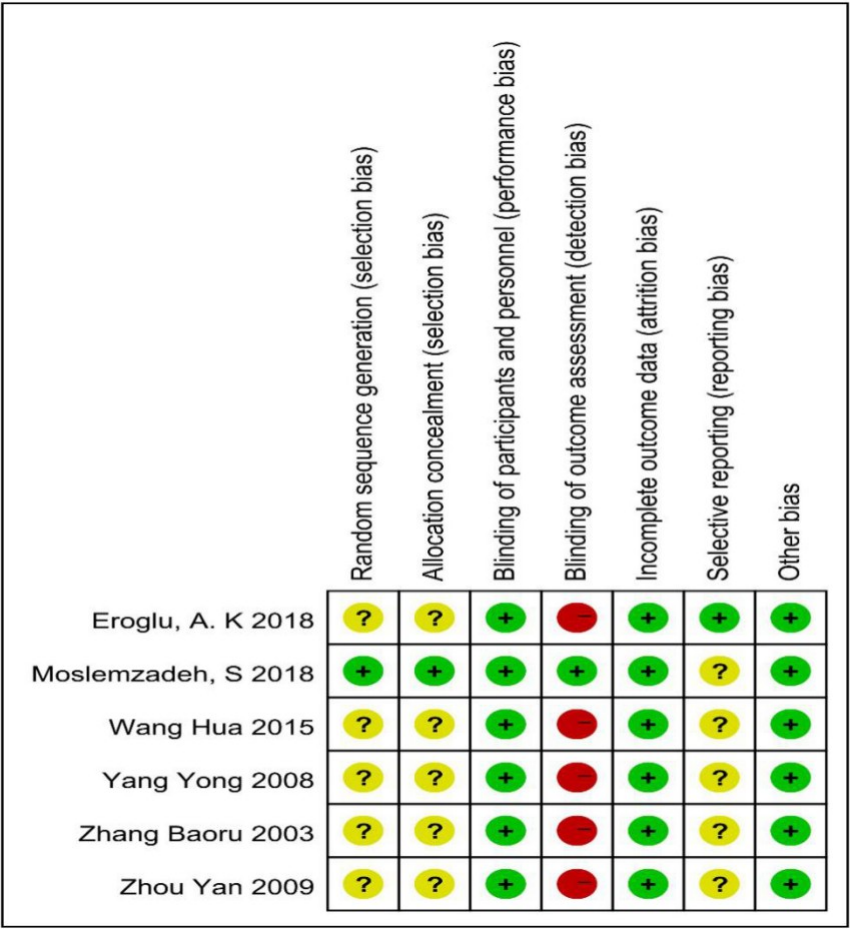
Summary of risk of bias.

**Fig 3:**
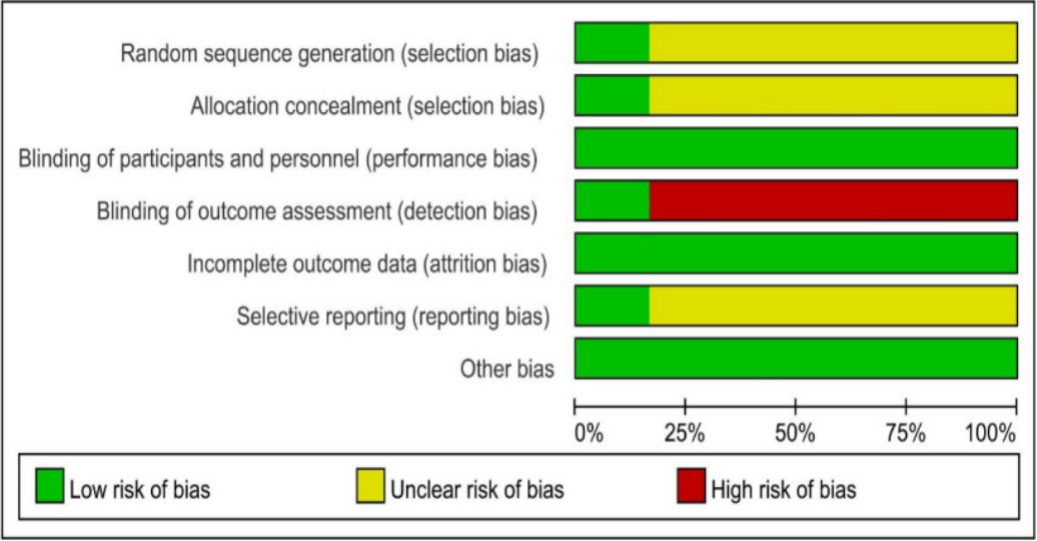
Graph depicting the risk of bias assessment.

Figs 2 and 3 show the bias risk summary plot and the bias risk plot, respectively. One trial was classified as having an ambiguous risk of bias. Five studies were classified as having a high risk of bias in the blinding of study outcomes.

### 3.3 The status of GI

**Fig 4:**
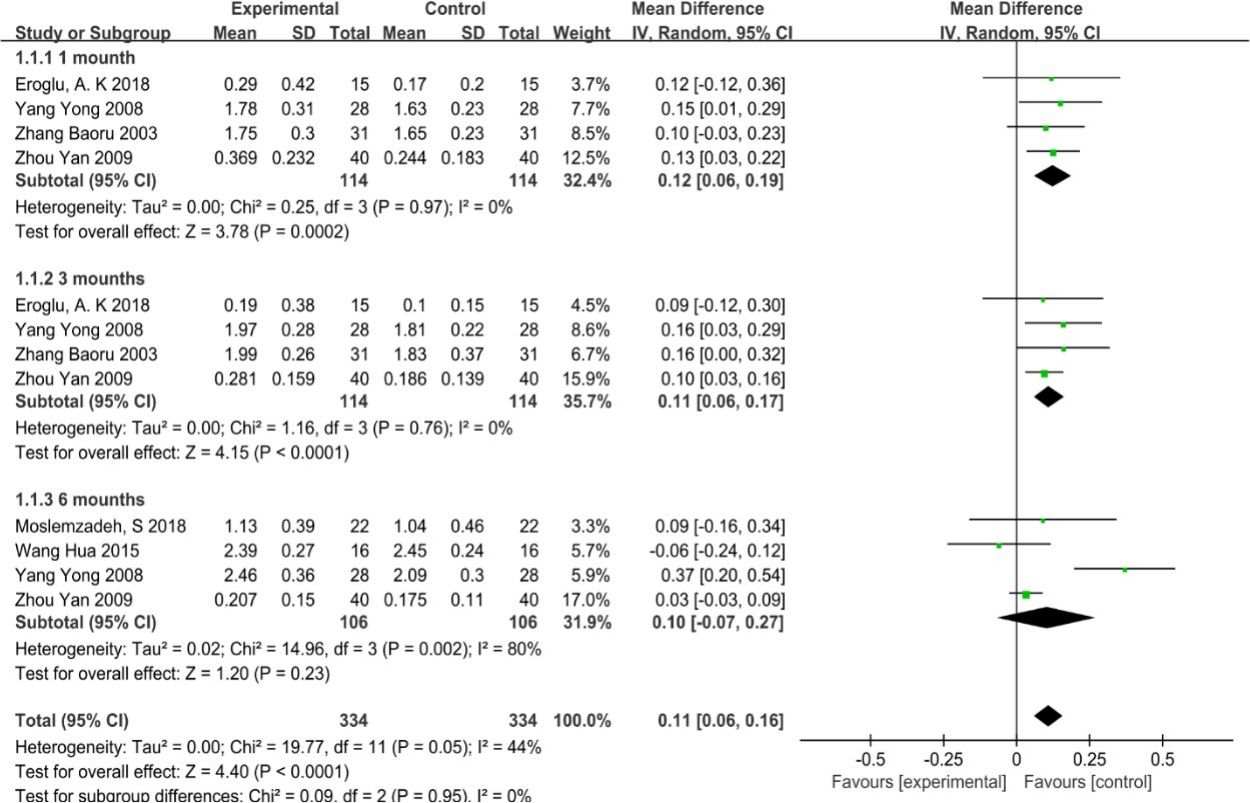
The status of GI index at 1, 3, and 6 months between the VFR group and the Hawley group.

**Fig 5:**
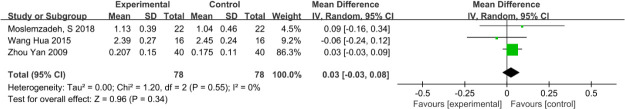
The status of GI index at 6 months between the VFR group and the Hawley group after excluding a large heterogeneity study.

The results of the meta-analysis (random-effects model) showed that there were significant differences in GI between the VFR group and the Hawley group, including at 1 month (mean difference = 0.12, 95%CI: 0.06 to 0.19) and 3 months (mean difference = 0.11, 95%CI: 0.06 to 0.17). At 6 months (mean difference = 0.10, 95% CI: -0.07 to 0.27), there was no significant difference. As shown in Fig 4, there was a great heterogeneity in GI at the 6th month (I2 = 80%). The greater heterogeneity may be due to Yong Yang’s subjective reasons at the time of measurement. As shown in Fig 5, heterogeneity was significantly reduced after excluding the study conducted by Yong Yang (mean difference = 0.03, 95%CI: -0.03 to 0.08, I2 = 0%).

### 3.4 Plaque index status

**Fig 6:**
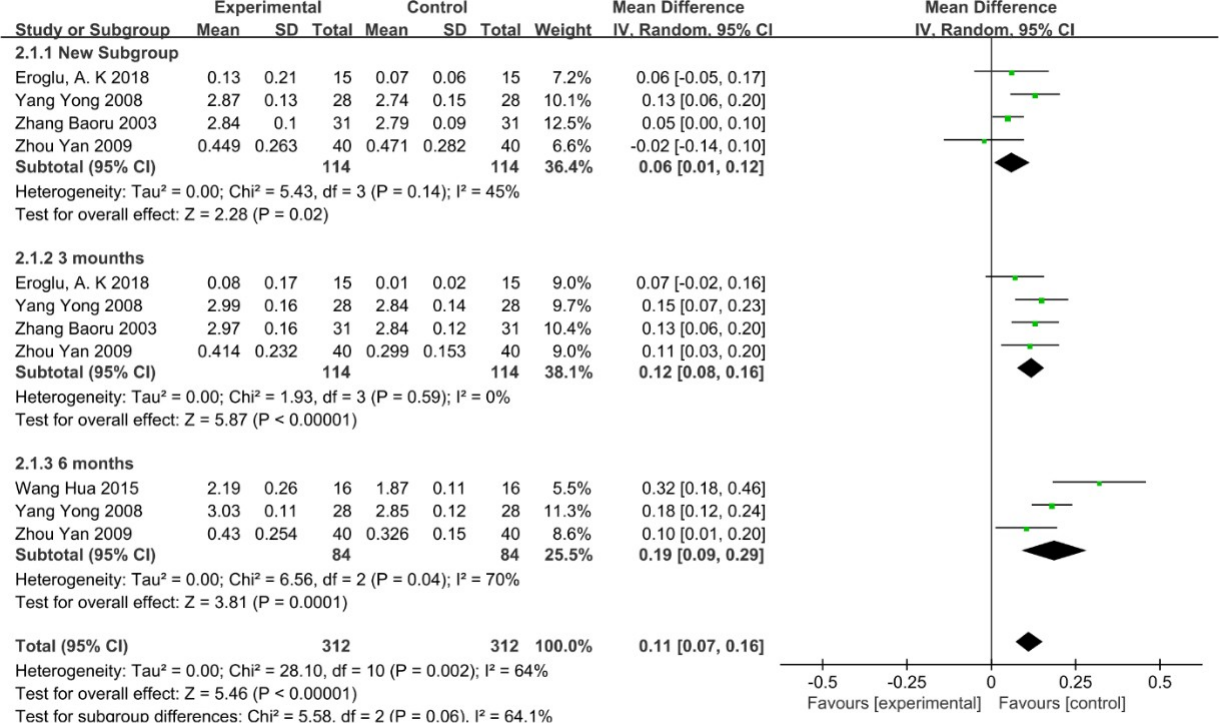
The status of PLI index at 1, 3, and 6 months between the VFR group and the Hawley group.

The results of the meta-analysis (random-effects model) showed that the PLI was significantly higher in the VFR group, including at 1 month (mean difference = 0.06, 95% CI: 0.01 to 0.12), 3 months (mean difference = 0.12, 95%CI: 0.08 to 0.16), and 6 months (mean difference = 0.19, 95%CI: 0.09 to 0.29), as shown in Fig 6. However, the PLI showed great heterogeneity at 6 months (I2 = 70%).

### 3.5 Subgroup of the status of PLI at 6 months

**Fig 7:**
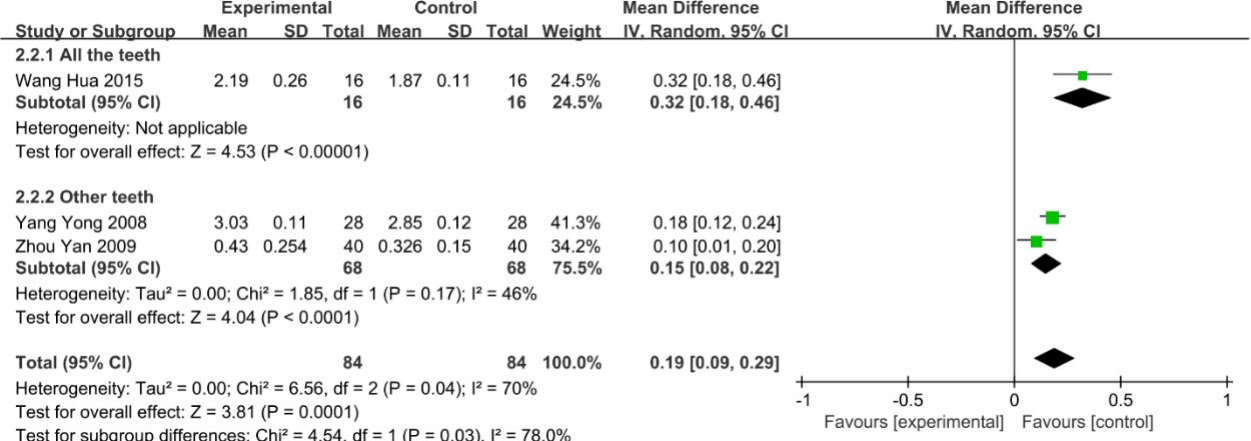
Subgroup of the status of PLI index at 6 months.

After a careful examination of each study, it was found that the included studies measured different tooth areas at 6 months. Wang Hua’s study measured PLI for all teeth, while the other two studies measured PLI for a total of 6 teeth: 11, 23, 26, 31, 43, and 46. Thus, a subgroup analysis was performed after dividing the patients into all-teeth groups and other teeth groups. The PLI of all teeth groups was calculated (mean difference = 0.32, 95%CI: 0.18 to 0.46), while the PLI of other teeth groups was (mean difference = 0.15, 95%CI: 0.08 to 0.22), and heterogeneity was significantly reduced (I2 = 46%), as shown in Fig 7.

### 3.6 Measurement of SPD

**Fig 8:**
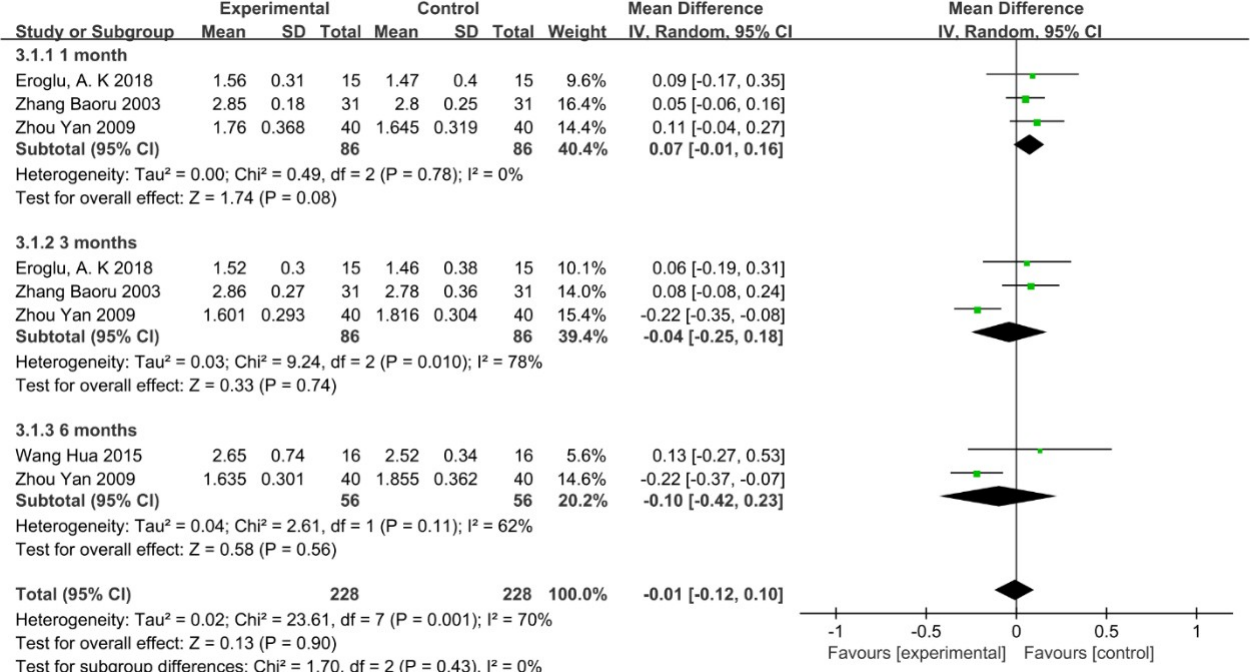
The status of SPD index at 1, 3, and 6 months between the VFR group and the Hawley group.

There was no significant difference in SPD between the VFR group and the Hawley group at 1 month (mean difference = 0.07,95% CI: -0.01 to 0.16), 3 months (mean difference = -0.04, 95% CI: -0.25 to 0.18), or 6 months (mean difference = -0.10, 95% CI: -0.42 to 0.23), as shown in Fig 8.

## 4. Discussion

Currently, vacuum-formed retainers (VFR) and Hawley retainer (HR) are the most commonly used removable retainers in clinical use [19]. Because VFR consist of a vacuum-formed thermal plastic holder made of PVC, it usually covers the entire dental arch, including the chewing surface of the tooth, and is popular among doctors and patients because of its transparency and esthetic appeal [20]. However, no consensus has been reached regarding the effect of VFR and HR on periodontal health. Groosh et al. [21] found that both VFR and HR could induce changes in the oral flora and increase the risk of periodontal disease compared with a blank group. Ristic et al. [22] pointed out that the first 6 months of using the orthodontic appliance represent the peak stage of gingivitis development, and then it gradually tends to maintain. Therefore, our study compared the periodontal conditions in the first 6 months, and the results showed that the Hawley group was significantly better than the VFR group in terms of GI and PLI. However, there was no significant difference in SPD score between the two groups. The findings from this meta-analysis suggest that Hawley retainers are better for periodontal health than vacuum-formed retainers.

This result may be mainly attributed to the following reasons. First, because VFR are wrapped around all the tooth surfaces for a long time, the self-cleaning effect of saliva on the tooth surface is seriously affected, and the residue and soft scale on the tooth surface and gingival crevicular could not be washed, resulting in plaque accumulation and irritation of the gingiva. Second, VFR is mainly made of polyvinyl chloride (PVC), and thus wear and scratches can easily increase its surface roughness, which along with its uneven structure, is conducive to bacterial attachment and biofilm formation. Studies have also shown that the formation of bacterial biofilms also depends on the physical and chemical properties of the material [23]. Finally, patients wearing HR can perform simple hygiene measures during the wearing period, such as the use of toothpick and dental floss, while VFR does not allow such hygiene measures.

Russell et al. [24] pointed out that the periodontal index, which is widely used in clinical practice, can objectively reflect the periodontal state. Our analysis shows that for GI status, the HR group has an advantage over the VFR group in the first three months. Significant heterogeneity was found at the sixth month. After a careful evaluation of the study by Yang Yong et al. [17], it was found that the authors did not explain in detail how the measurements were carried out. After excluding a study with high heterogeneity, we found no statistical difference in GI between the two groups at sixth months. This may be because, as found in some studies [10], the first three months of wearing retainers pose a higher risk of plaque accumulation and changes in periodontal bacteria, while the internal environment of oral flora tends to be stable when the retainers reach the sixth month.

As shown in Fig 6, our study concluded that in terms of PLI, there were significant differences between the VFR group and the HR group at 1, 3, and 6 months, and the VFR group was significantly higher than the HR group. In fact, this difference was obvious. The PLI was calculated as the area covered by plaque on the tooth surface. VFR covers all the surfaces of teeth, which is conducive to the colonization of anaerobes in the oral cavity and the increase of plaque. As stated in the study of Qian Wang et al. [25], the use of VFR covering all dental surfaces causes the imbalance of oral microorganisms and affects periodontal health. As for the high heterogeneity of PLI at the sixth month, we reevaluated the included studies and found that this occurred because, in Wang Hua’s study [16], all teeth were measured, while only representative teeth were measured in other studies. After subgroup analysis, it was shown that the HR group was significantly better than the VFR group, as shown in Fig 7.

SPD is the main method for the clinical evaluation of the degree of loss of periodontal supporting tissue. When periodontitis is present, combined with epithelial retreat to the gingival side, loss of attachment occurs, the probing depth increases, and the formation of the periodontal pocket can be determined [26]. This meta-analysis showed no significant differences between the VFR and Hawley groups at 1, 3, and 6 months. This may be because of the use of the retainer in the presence of increased unfavorable periodontal factors. However, the use of the retainer did not cause the loss of periodontal attachment. Thus, the depth of the two groups of did not show a great change, and no obvious changes were observed in the longitudinal comparison either. However, we found a significant heterogeneity in our study at the third and sixth months, which may have some effect. The main factors leading to heterogeneity are manifold, including different dental positions or different measurement techniques. Due to the limited number of studies, we do not provide a detailed explanation here. Miethke and Vogt [27] measured the SPD scores of fixed appliances and invisible appliances at different time points, and found no statistically significant difference in SPD between the two groups, which was consistent with our results. The use of VFR and HR only increased periodontal sensitivity, but did not directly cause periodontitis.

In recent years, attention has been paid to periodontitis, and evidence has shown that the older you are, the more likely you are to develop periodontal disease [28]. The use of orthodontics is considered to be the predisposing factor of periodontal disease during orthodontics, and the use of retainers may interfere with oral hygiene procedures and lead to plaque accumulation [22]. Some studies have also shown that if plaque is not well controlled, it can cause inflammation in patients, leading to the occurrence of periodontitis and loss of attachment [29, 30]. However, maintaining the long-term stability of treatment outcomes in patients who have completed orthodontic treatment is a great challenge for orthodontists and patients, especially the recurrence of anterior tooth congestion, which has been frequently reported during the follow-up after orthodontic treatment [31]. Therefore, long-term wear of retainers is necessary. Compared with Hawley retainers, vacuum-formed retainers are thinner and more comfortable in appearance. Some patients with a higher esthetic demand are more willing to accept such retainers. Licia Manzon et al. [32] also found that the concavity, scratches, and wear on the surface of VFR increased the surface roughness, which was more conducive to the colonization of bacteria. The percentage of bleeding sites increased in patients wearing VFR, which also confirmed the gingival problem, in agreement with our research results. Hawley retainer has some influence on the pronunciation of patients in the early stage, and the lip steel wire affects the patient’s appearance [33]. However, it still has some advantages in terms of periodontal health. From the perspective of clinical periodontal index, plaque (which causes adverse effects on gingival health) was more likely to form in users of vacuum-formed retainers than in users of the Hawley retainers. However, no significant difference was found in terms of retention effect [34]. The use of retainers is only one of the factors that increase the risk of periodontitis, and it does not directly cause periodontitis. The most important aspect is good oral hygiene procedures. People with good oral hygiene can effectively control periodontal tissue inflammation [35].

The limitations of this meta-analysis are as follows: some studies used small sample sizes, which may cause bias to a certain extent. Periodontal index for the position of the teeth was not determined. Further, some studies assessed individual teeth, whereas others assessed all the teeth. The included studies were mainly from China, Turkey, and Iran, and there is a lack of relevant studies from other world regions. Due to the language limitation of the included study, only articles in English and Chinese were included, leading to the exclusion of some articles in other languages, such as French, which will increase the publication bias. The specific structure of both types of orthodontic retainers is not clear, and there may be confounding factors. There is still a need for more high-quality RCTs due to the paucity of literature on the relationship between periodontitis and orthodontic maintenance.

## 5. Conclusion

Our meta-analysis showed that patients using Hawley retainers showed advantages in terms of periodontal health. Hawley retainers also showed a lower adverse effect on periodontal tissue compared with vacuum-formed retainers. However, due to the limited number and quality of studies on this topic, this conclusion needs to be confirmed by more high-quality studies.

## Supporting information

S1 Table The characteristics of the excluded studies. (DOC)

S1 Checklist PRISMA checklist. (DOC)

S1 File Data availability. (DOCX)

## Abbreviations

95%CI: 95% confidence interval
RCT: randomized controlled trial
GI: gingival index
PLI: plaque index
SPD: sulcus probing depth
VFR: vacuum-formed retainer
HR: Hawley retainer

## References

[1] Cerny R., Cockrell D., and Lloyd D., A survey of patient opinions on fixed vs. removable retainers. J Clin Orthod, 2009. 43(12): p. 784–7. 20391855

[2] Johnston C.D. and Littlewood S.J., Retention in orthodontics. Br Dent J, 2015. 218(3): p. 119–22. doi: 10.1038/sj.bdj.2015.47 25686428

[3] Horowitz S.L. and Hixon E.H., Physiologic recovery following orthodontic treatment. Am J Orthod, 1969. 55(1): p. 1–4. doi: 10.1016/s0002-9416(69)90168-7 5248054

[4] Blake M. and Bibby K., Retention and stability: a review of the literature. Am J Orthod Dentofacial Orthop, 1998. 114(3): p. 299–306. doi: 10.1016/s0889-5406(98)70212-4 9743135

[5] Sun J., et al., Survival time comparison between Hawley and clear overlay retainers: a randomized trial. J Dent Res, 2011. 90(10): p. 1197–201. doi: 10.1177/0022034511415274 21771797

[6] Barlin S., et al., A retrospective randomized double-blind comparison study of the effectiveness of Hawley vs vacuum-formed retainers. Angle Orthod, 2011. 81(3): p. 404–9. doi: 10.2319/072610-437.1 21261482 PMC8923547

[7] Kaklamanos E.G., et al., Performance of clear vacuum-formed thermoplastic retainers depending on retention protocol: a systematic review. Odontology, 2017. 105(2): p. 237–247. doi: 10.1007/s10266-016-0254-5 27270920

[8] Jäderberg S., Feldmann I., and Engström C., Removable thermoplastic appliances as orthodontic retainers—a prospective study of different wear regimens. Eur J Orthod, 2012. 34(4): p. 475–9. doi: 10.1093/ejo/cjr040 21508267

[9] Shawesh M., et al., Hawley retainers full- or part-time? A randomized clinical trial. Eur J Orthod, 2010. 32(2): p. 165–70. doi: 10.1093/ejo/cjp082 19797411

[10] Batoni G., et al., Effect of removable orthodontic appliances on oral colonisation by mutans streptococci in children. Eur J Oral Sci, 2001. 109(6): p. 388–92. doi: 10.1034/j.1600-0722.2001.00089.x 11767275

[11] Addy M., et al., The effect of orthodontic appliances on the distribution of Candida and plaque in adolescents. Br J Orthod, 1982. 9(3): p. 158–63. doi: 10.1179/bjo.9.3.158 6954991

[12] Eroglu A.K., Baka Z.M., and Arslan U., Comparative evaluation of salivary microbial levels and periodontal status of patients wearing fixed and removable orthodontic retainers. Am J Orthod Dentofacial Orthop, 2019. 156(2): p. 186–192. doi: 10.1016/j.ajodo.2018.08.022 31375228

[13] Zhang BR and Wang QM, Periodontal implication of positioner versus removable retainer Beijing J Stom, 2003(03): p. 146–147+155.

[14] Moher D., et al., Preferred reporting items for systematic reviews and meta-analyses: the PRISMA Statement. Open Med, 2009. 3(3): p. e123–30. 21603045 PMC3090117

[15] Moslemzadeh S.H., et al., Comparison of Stability of the Results of Orthodontic Treatment and Gingival Health between Hawley and Vacuum-formed Retainers. J Contemp Dent Pract, 2018. 19(4): p. 443–449. 29728551

[16] Wang H, Ling MR, and Ling S, The effect of three different orthodontic retainers on gingival crevicular fluid. J of Modern Stomatology, 2015. 29(04): p. 220–222.

[17] Yang Y and Hu YH, The effect of two kinds of retainers on periodontal health. Proceeding of Clinical Medicine, 2008(09): p. 745–746.

[18] Zhou Y, Fang ZX, and Wei HP, Periodontal health care of patients wearing the reformed transparent full-arch wraparound retainers and the effects of the oral hygiene care during maintenance periods. Chinese J of New Clinical Medicine, 2009. 2(04): p. 370–373.

[19] Mai W., et al., Comparison of vacuum-formed and Hawley retainers: a systematic review. Am J Orthod Dentofacial Orthop, 2014. 145(6): p. 720–7. doi: 10.1016/j.ajodo.2014.01.019 24880842

[20] Kumar A.G. and Bansal A., Effectiveness and acceptability of Essix and Begg retainers: a prospective study. Aust Orthod J, 2011. 27(1): p. 52–6. 21696115

[21] Al Groosh D., et al., The prevalence of opportunistic pathogens associated with intraoral implants. Lett Appl Microbiol, 2011. 52(5): p. 501–5. doi: 10.1111/j.1472-765X.2011.03031.x 21332760

[22] Ristic M., et al., Clinical and microbiological effects of fixed orthodontic appliances on periodontal tissues in adolescents. Orthod Craniofac Res, 2007. 10(4): p. 187–95. doi: 10.1111/j.1601-6343.2007.00396.x 17973685

[23] Kusuma Yulianto H.D., et al., Biofilm composition and composite degradation during intra-oral wear. Dent Mater, 2019. 35(5): p. 740–750. doi: 10.1016/j.dental.2019.02.024 30833012

[24] Russell A.L., The Periodontal Index. J Periodontol, 1967. 38(6): p. Suppl:585–91. doi: 10.1902/jop.1967.38.6_part2.585 5237681

[25] Wang Q., et al., Alterations of the oral microbiome in patients treated with the Invisalign system or with fixed appliances. Am J Orthod Dentofacial Orthop, 2019. 156(5): p. 633–640. doi: 10.1016/j.ajodo.2018.11.017 31677672

[26] Listgarten M.A., Periodontal probing: what does it mean? J Clin Periodontol, 1980. 7(3): p. 165–76. doi: 10.1111/j.1600-051x.1980.tb01960.x 7000852

[27] Miethke R.R. and Vogt S., A comparison of the periodontal health of patients during treatment with the Invisalign system and with fixed orthodontic appliances. J Orofac Orthop, 2005. 66(3): p. 219–29. doi: 10.1007/s00056-005-0436-1 15959635

[28] Gkantidis N., Christou P., and Topouzelis N., The orthodontic-periodontic interrelationship in integrated treatment challenges: a systematic review. J Oral Rehabil, 2010. 37(5): p. 377–90. doi: 10.1111/j.1365-2842.2010.02068.x 20202098

[29] Artun J. and Urbye K.S., The effect of orthodontic treatment on periodontal bone support in patients with advanced loss of marginal periodontium. Am J Orthod Dentofacial Orthop, 1988. 93(2): p. 143–8. doi: 10.1016/0889-5406(88)90292-2 3422529

[30] Wennström J.L., et al., Periodontal tissue response to orthodontic movement of teeth with infrabony pockets. Am J Orthod Dentofacial Orthop, 1993. 103(4): p. 313–9. doi: 10.1016/0889-5406(93)70011-C 8480696

[31] Steinnes J., Johnsen G., and Kerosuo H., Stability of orthodontic treatment outcome in relation to retention status: An 8-year follow-up. Am J Orthod Dentofacial Orthop, 2017. 151(6): p. 1027–1033. doi: 10.1016/j.ajodo.2016.10.032 28554448

[32] Manzon L., et al., Periodontal health and compliance: A comparison between Essix and Hawley retainers. Am J Orthod Dentofacial Orthop, 2018. 153(6): p. 852–860. doi: 10.1016/j.ajodo.2017.10.025 29853243

[33] Atik E., et al., Comparing the effects of Essix and Hawley retainers on the acoustics of speech. Eur J Orthod, 2017. 39(4): p. 440–445. doi: 10.1093/ejo/cjw050 27507127

[34] Demir A., et al., Comparison of retention characteristics of Essix and Hawley retainers. Korean J Orthod, 2012. 42(5): p. 255–62. doi: 10.4041/kjod.2012.42.5.255 23173119 PMC3495257

[35] van Gastel J., et al., Longitudinal changes in microbiology and clinical periodontal parameters after removal of fixed orthodontic appliances. Eur J Orthod, 2011. 33(1): p. 15–21. doi: 10.1093/ejo/cjq032 20671070

