# Supplementary material for: Assessment of the effect of vacuum-formed retainers and Hawley retainers on periodontal health: A systematic review and meta-analysis
Source: PLoS One. 2021 Jul 9;16(7):e0253968. doi: 10.1371/journal.pone.0253968 (PMC8270199; doi:10.1371/journal.pone.0253968)
Supplement: S1 Table — (DOC) [file pone.0253968.s001.doc]

| Reference  **S1 Table**. **List of all studies selected for full text analysis and reasons for exclusion.** | Classification |
| --- | --- |
| 1. Ahn, S.-J., et al., *Changes in salivary periodontal pathogens after orthodontic treatment: An in vivo prospective study.* The Angle Orthodontist, 2016. **86**(6): p. 998-1003. | NOT SELECTED. Not randomized controlled study. |
| 1. Alrawas, M.B., et al., *Comparing the effects of CAD/CAM nickel-titanium lingual retainers on teeth stability and periodontal health with conventional fixed and removable retainers: A randomized clinical trial.* Orthodontics & Craniofacial Research, 2020. **31**: p. 31. | NOT SELECTED. Describe the other retainers. |
| 1. Arn, M.L., et al., *The effects of fixed orthodontic retainers on periodontal health: A systematic review.* American Journal of Orthodontics and Dentofacial Orthopedics, 2020. **157**(2): p. 156-+. | NOT SELECTED. Review. |
| 1. Barlin, S., et al., *A retrospective randomized double-blind comparison study of the effectiveness of Hawley vs vacuum-formed retainers.* Angle Orthod, 2011. **81**(3): p. 404-9. | NOT SELECTED. Not randomized controlled study. |
| 1. Batoni, G., et al., *Effect of removable orthodontic appliances on oral colonisation by mutans streptococci in children.* Eur J Oral Sci, 2001. **109**(6): p. 388-92. | NOT SELECTED. Other retainers. |
| 1. Blake, M. and K. Bibby, *Retention and stability: a review of the literature.* Am J Orthod Dentofacial Orthop, 1998. **114**(3): p. 299-306. | NOT SELECTED. Not randomized controlled study. |
| 1. Cerny, R., D. Cockrell, and D. Lloyd, *A survey of patient opinions on fixed vs. removable retainers.* J Clin Orthod, 2009. **43**(12): p. 784-7. | NOT SELECTED. Other retainers. |
| 1. Chhibber, A., et al., *Which orthodontic appliance is best for oral hygiene? A randomized clinical trial.* Am J Orthod Dentofacial Orthop, 2018. **153**(2): p. 175-183. | NOT SELECTED. Other retainers. |
| 1. Cifter, M., A.D.G. Celikel, and A. Cekici, *Effects of vacuum-formed retainers on periodontal status and their retention efficiency.* American Journal of Orthodontics and Dentofacial Orthopedics, 2017. **152**(6): p. 830-835. | NOT SELECTED. Not randomized controlled study. |
| 1. D, D.I.V., et al., *Correlation between parodontal indexes and orthodontic retainers: prospective study in a group of 16 patients.* Oral Implantol (Rome), 2017. **10**(1): p. 78-86. | NOT SELECTED. Not randomized controlled study. |
| 1. Manzon, L., et al., *Periodontal health and compliance: A comparison between Essix and Hawley retainers.* Am J Orthod Dentofacial Orthop, 2018. **153**(6): p. 852-860. | NOT SELECTED. Statistical data unavailable. |
| 1. Gelin, E., et al., *Innovative customized CAD/CAM nickel-titanium lingual retainer versus standard stainless-steel lingual retainer: A randomized controlled trial.* The Korean journal of orthodontics, 2020. **50**(6): p. 373-382. | NOT SELECTED. Other retainers. |
| 1. Gökçe, B. and B. Kaya, *Periodontal effects and survival rates of different mandibular retainers: comparison of bonding technique and wire thickness.* Eur J Orthod, 2019. **41**(6): p. 591-600. | NOT SELECTED. Not randomized controlled study. |
| 1. Heier, E.E., et al., *Periodontal implications of bonded versus removable retainers.* Am J Orthod Dentofacial Orthop, 1997. **112**(6): p. 607-16. | NOT SELECTED. Other retainers. |
| 1. Jäderberg, S., I. Feldmann, and C. Engström, *Removable thermoplastic appliances as orthodontic retainers--a prospective study of different wear regimens.* Eur J Orthod, 2012. **34**(4): p. 475-9. | NOT SELECTED. Not randomized controlled study. |
| 1. Knaup, I., et al., *Potential impact of lingual retainers on oral health: comparison between conventional twistflex retainers and CAD/CAM fabricated nitinol retainers: Aclinical in vitro and in vivo investigation.* Journal of Orofacial Orthopedics-Fortschritte Der Kieferorthopadie, 2019. **80**(2): p. 88-96. | NOT SELECTED. Other retainers. |
| 1. Rody, W.J., Jr., et al., *Effects of different orthodontic retention protocols on the periodontal health of mandibular incisors.* Orthod Craniofac Res, 2016. **19**(4): p. 198-208. | NOT SELECTED. Statistical data unavailable. |
| 1. Al-Hamdany, A.K., A.R. Al-Khatib, and H.I. Al-Sadi, *Influence of olive oil on alveolar bone response during orthodontic retention period: rabbit model study.* Acta Odontol Scand, 2017. **75**(6): p. 413-422. | NOT SELECTED. Animal  Study. |
| 1. Cacoub, M. and C. Chabre, *Contentions collées orthodontiques et impact parodontal*. 2013. | NOT SELECTED. Language limitation |
