# Supplementary material for: Assessment of the effect of vacuum-formed retainers and Hawley retainers on periodontal health: A systematic review and meta-analysis
Source: PLoS One. 2021 Jul 9;16(7):e0253968. doi: 10.1371/journal.pone.0253968 (PMC8270199; doi:10.1371/journal.pone.0253968)
Supplement: S1 Checklist — (DOC) [file pone.0253968.s002.doc]

**Screening**

**Included**

**Eligibility**

**Identification**

Records identified through database searching
(n =201 )

Additional records identified through other sources
(n = 1 )

Records after duplicates removed
(n = 46 )

Records screened
(n = 46 )

Records excluded
(n =21 )

Full-text articles assessed for eligibility
(n = 25 )

Full-text articles excluded, with reasons
(n = 19 )

1. Non-randomised studies(n=8)
2. Unavailable for date extraction(n=2)
3. Animal studies (n=1)
4. Other retainers(n=7)
5. Language limitation(n=1)

Studies included in qualitative synthesis
(n =6 )

Studies included in quantitative synthesis (meta-analysis)
(n =6 )
