# Supplementary material for: Assessment of the effect of vacuum-formed retainers and Hawley retainers on periodontal health: A systematic review and meta-analysis
Source: PLoS One. 2021 Jul 9;16(7):e0253968. doi: 10.1371/journal.pone.0253968 (PMC8270199; doi:10.1371/journal.pone.0253968)
Supplement: S1 File — (DOCX) [file pone.0253968.s003.docx]

**Table1.** **Original data of Yang Yong**

| Indicator | Time | VFR | Hawley |
| --- | --- | --- | --- |
| GI | baseline  1 month  3month  6month | 1.52±0.23  1.78±0.31  1.97±0.28  2.46±0.36 | 1.56±0.24  1.63±0.23  1.81±0.22  2.09±0.30 |
| PLI | baseline  1 month  3month  6month | 2.60±0.10  2.87±0.13  2.99±0.16  3.03±0.11 | 2.57±0.11  2.74±0.15  2.84±0.14  2.85±0.12 |

**Table2. Original data of Zhang Baoru**

| Indicator | Time | VFR | Hawley |
| --- | --- | --- | --- |
| PD | baseline  1 month  3month  6month | 2.59±0.22  2.85±0.18  2.86±0.27  NM | 2.50±0.31  2.80±0.25  2.78±0.36  NM |
| PLI | baseline  1 month  3month  6month | 2.60±0.12  2.84±0.10  2.97±0.16  NM | 2.56±0.10  2.79±0.09  2.84±0.12  NM |
| GI | baseline  1 month  3month  6month | 1.50±0.31  1.75±0.30  1.99±0.26  NM | 1.56±0.21  1.65±0.23  1.83±0.37  NM |

**Table3. Original data of Zhou Yan**

| Indicator | Time | VFR | Hawley |
| --- | --- | --- | --- |
| PD | baseline  1 month  3month  6month | 1.502±0.390  1.760±0.368  1.601±0.293  1.635±0.301 | 1.696±0.349  1.645±0.319  1.816±0.304  1.855±0.362 |
| PLI | baseline  1 month  3month  6month | 0.057±0.037  0.449±0.263  0.414±0.232  0.430±0.254 | 0.075±0.128  0.471±0.282  0.299±0.153  0.326±0.150 |
| GI | baseline  1 month  3month  6month | 0.364±0.258  0.369±0.232  0.281±0.159  0.207±0.150 | 0.271±0.184  0.244±0.183  0.186±0.139  0.175±0.110 |

**Table4. Original data of Wang Hua**

| Indicator | Time | VFR | Hawley |
| --- | --- | --- | --- |
| PD | baseline  1 month  3month  6month | 1.90±0.33  NM  NM  2.65±0.74 | 1.87±0.32  NM  NM  2.52±0.34 |
| PLI | baseline  1 month  3month  6month | 0.13±0.15  NM  NM  2.19±0.26 | 0.12±0.22  NM  NM  1.87±0.11 |
| GI | baseline  1 month  3month  6month | 1.53±0.65  NM  NM  2.39±0.27 | 1.56±0.42  NM  NM  2.45±0.24 |

**Table5. Original data of Eroglu, A. K**

| Indicator | Time | VFR | Hawley |
| --- | --- | --- | --- |
| PD | baseline  1 month  3month  6month | 1.81±0.36  1.56±0.31  1.52±0.30  NM | 1.76±0.39  1.47±0.40  1.46±0.38  NM |
| PLI | baseline  1 month  3month  6month | 0.60±0.44  0.23±0.27  0.08±0.17  NM | 0.50±037  0.07±0.06  0.01±0.02  NM |
| GI | baseline  1 month  3month  6month | 1.53±0.65  NM  NM  2.39±0.27 | 1.56±0.42  NM  NM  2.45±0.24 |

**Table6. Original data of Moslemzadeh, S. H**

| Indicators | Time | VFR | Hawley |
| --- | --- | --- | --- |
| PD | baseline  1 month  3month  6month | NM  NM  NM  NM | NM  NM  NM  NM |
| PLI | baseline  1 month  3month  6month | NM  NM  NM  NM | NM  NM  NM  NM |
| GI | baseline  1 month  3month  6month | 1.43±0.31  NM  NM  1.19±0.44 | 1.33±0.27  NM  NM  1.04±0.46 |

**NM : not mention Mean±SD(mm)**
